# Comprehensive Nutritional Profiling of Kernels in 141 Apricot Cultivars Based on Amino Acids, Fatty Acids, Vitamins, and Mineral Elements

**DOI:** 10.3390/foods15203588

**Published:** 2026-10-09

**Authors:** Junhuan Zhang, Wenjian Yu, Meiling Zhang, Li Yang, Fengchao Jiang, Boris Krška, Haoyuan Sun

**Affiliations:** 1 Institute of Forestry and Pomology, Beijing Academy of Agriculture and Forestry Sciences, Beijing 100093, China; 2 Key Laboratory of National Forestry and Grassland Administration on Apricot, Beijing 100093, China; 3 Key Laboratory of Urban Agriculture (North China), Ministry of Agriculture and Rural Affairs, Beijing 100093, China; 4 Research and Breeding Institute of Pomology Holovousy Ltd., Holovousy 129, 508 01 Holovousy, Czech Republic

**Keywords:** apricot kernel, nutritional quality, genetic diversity, comprehensive evaluation

## Abstract

Apricot kernels are nutritionally valuable and possess both edible and medicinal properties. However, there is little published data on the differences in kernel nutrition among a large number of cultivars, which has limited the development and utilization of specialty apricot processed products. In this study, 15 nutritional components were measured in kernels of 141 apricot cultivars, followed by comprehensive statistical analyses including genetic diversity, principal component, correlation, and cluster analyses, along with a comprehensive evaluation. The genetic diversity index (*H*′) for all indicators exceeded 1.0, with fat, protein, oleic acid, linoleic acid, and vitamin E showing *H*′ > 2.0, indicating nutritional diversity among cultivars. Correlation analysis revealed that fat content was significantly positively correlated with oleic acid, palmitic acid, and total fatty acids (r > 0.7), while oleic acid was negatively correlated with vitamin E and γ-tocopherol. The first five principal components accounted for 75.614% of the total variance, effectively capturing the essential information of the 15 traits. Cluster analysis at a Euclidean distance of 19.5 divided the 141 accessions into five groups, each with distinctive nutritional profiles. Based on the comprehensive evaluation of 15 nutritional components, the apricot cultivar ‘Kuhe Honglian’ achieved the highest score, followed by ‘Machuanling’, ‘Guajiayutianhexiangbai’, ‘Guanlaoyelian’, and ‘Graflthan’. These findings provide a foundation for advancing the apricot kernel processing industry and supporting the breeding of functional kernel-apricot varieties.

## 1. Introduction

Apricot (*Prunus armeniaca*) is an economically significant fruit tree within the genus *Prunus* (family *Rosaceae*). It is renowned for its distinctive fruit flavor and nutrient-rich kernels. The apricot tree is also characterized by its tolerance to poor soils, drought resistance, and high productivity, attributes that have facilitated its widespread cultivation globally. In China, the cultivation history spans 3000 to 4000 years, accompanied by exceptionally rich germplasm, including more than 2000 varieties and types [1]. Historically, research and development within apricot production have predominantly focused on fresh-market or processing fruits, emphasizing traits such as flesh flavor, color, firmness, and postharvest preservation [2,3,4]. However, the apricot kernel, the seed contained within the stone and a primary by-product of fruit processing, has often been overlooked or discarded, resulting in significant resource waste. In fact, apricot kernels are rich in various nutrients and not only serve as a high-quality nut for consumption but also possess considerable medicinal and health properties, making them an ideal raw material for the development of functional foods and pharmaceuticals [5,6,7]. The nutritional value of apricot kernels is primarily attributed to their abundant oil, protein, vitamin E, and diverse mineral elements. The kernel oil is rich in unsaturated fatty acids, particularly oleic and linoleic acids, which play a positive role in reducing the risk of cardiovascular diseases and regulating blood lipids [8,9,10]. The kernel protein contains various essential amino acids for humans, while vitamin E, a natural fat-soluble antioxidant, demonstrates significant efficacy in delaying aging and enhancing immune function [11,12,13]. Notably, the nutritional composition of apricot kernels varies considerably among different cultivars [14]. Therefore, a systematic evaluation of the nutritional quality of kernels across diverse apricot germplasm resources is of great significance for fully realizing the utilization potential of apricot kernels and enhancing the overall economic benefits of the apricot industry.

The variation among cultivars and comprehensive evaluations based on phenotypic traits in germplasm resources have been reported in various fruit tree species, including kiwifruit, pear, jujube, and pomegranate [15,16,17,18]. Common methods for screening key phenotypic traits and conducting comprehensive evaluations include principal component analysis, cluster analysis, correlation analysis, and membership function analysis [19,20,21]. The genetic diversity in apricot germplasm resources has also been analyzed based on phenotypic traits. The fruit acidity and organic acid profiles in the flesh and skin of 164 apricot accessions from northeastern Italy were investigated, revealing an exceptionally high level of diversity within European apricot germplasm [22]. Karataş et al. conducted the first detailed analysis of the morphological, nutritional, and nutraceutical properties of 14 selected wild apricot genotypes from Turkey [23]. The carotenoid composition in the peel and flesh of 41 apricot cultivars from Xinjiang was qualitatively and quantitatively assessed, with results indicating substantial metabolic diversity in apricot carotenoids [24]. To estimate broad-sense heritability for shell and kernel traits, phenotypic data from 222 accessions were evaluated across 19 traits, including kernel characteristics, stone shell properties, and pistil abortion rate [25]. Chen et al. performed a diversity assessment and evaluation of 165 wild Siberian apricot (*Prunus sibirica* L.) resources originating from the Yanshan–Taihang Mountain region in Hebei Province, China, using 21 fruit quality phenotypic traits. Through factor analysis, the original 21 traits were effectively reduced to a simplified seven-trait model, which accounted for over 80% of the total quality variation [26].

It is evident that previous studies mainly focused on the analysis of morphological traits of apricot germplasm, such as leaves, fruits, kernels, and shells, with limited coverage of the nutritional quality indicators of apricot kernels. Moreover, most studies targeted only partial regional varieties, primarily wild apricot germplasm, with few focusing mainly on cultivated varieties. Data on the nutritional quality of apricot kernels were even fewer. Hence, the objectives of this study were to investigate the nutritional characteristics of apricot kernels from domestic and international apricot cultivars. A total of 141 apricot cultivars were used as experimental materials, and fifteen nutritional components in apricot kernels, including protein, fat, various fatty acids, amino acids, vitamins, and minerals, were compared and assessed. The genetic diversity index and coefficient of variation were calculated. Correlation analysis, principal component analysis and cluster analysis were also employed to screen the top nutritional cultivars. This will provide a foundation for the scientific selection of elite germplasm with specific nutritional advantages (e.g., rich in unsaturated fatty acids and vitamin E). It would also offer important theoretical support for improving kernel processing and breeding functional varieties in apricot.

## 2. Materials and Methods

### 2.1. Plant Materials

The 141 apricot cultivars used in this study (Table 1) were collected from the apricot germplasm repository of the Beijing Academy of Forestry and Pomology Sciences. For each apricot cultivar, fruits were collected from three individual trees (three independent biological replicates). Thirty to fifty fruits were harvested from each tree. Kernels from each tree were separated, dried and ground separately; samples from different trees were not pooled. Each tree represented one biological replicate. Sampling was performed from early June to mid-July 2025. All cultivars were cultivated in the same orchard with identical soil, water, fertilizer and light conditions.

### 2.2. Determination of Protein and Fat

The protein content was determined by the Kjeldahl method according to GB 5009.5-2016 [27]. Briefly, apricot kernel powder (0.2000–0.5000 g) was accurately weighed into a digestion tube. A catalyst mixture (0.4 g of copper sulfate and 6 g of potassium sulfate) and 10 mL of concentrated sulfuric acid were added for mineralization. Samples were digested on a graphite digestion system with programmed heating: 150 °C for 30 min, 220 °C for 15 min, and then 420 °C for 120 min until the digest solution became clear blue-green. After cooling to room temperature, total nitrogen was measured using an automatic Kjeldahl nitrogen analyzer (e.g., K1100, Hanon Instruments, Jinan, Shandong, China). A nitrogen-to-protein conversion factor of 5.30 was applied to apricot kernel samples. Reagent blanks were run in parallel.

For fat determination, approximately 1.0 g of sample was weighed into a conical flask, and the mass was recorded as ‘m’. Ten milliliters of water and 10 mL of concentrated hydrochloric acid were added, mixed thoroughly, and the mixture was hydrolyzed in a 70–80 °C water bath for 30–40 min with continuous shaking. After cooling, 10 mL of ethanol was added and mixed. Extraction was performed three times, each with 25 mL of petroleum ether. The combined ether layers were dried over anhydrous sodium sulfate and transferred into a pre-weighed evaporating dish (m_0_). The solvent was evaporated in a water bath, and the residue was dried in an oven at 100 °C for 1 h, cooled, and weighed (m_1_). Crude fat (%) was calculated as [(m_1_ − m_0_)/m] × 100. All determinations were conducted in duplicate.

### 2.3. Extraction and Determination of Amino Acids

The contents of 18 amino acids in the samples were determined using an automatic amino acid analyzer (L-8900, Hitachi High-Tech Science Corporation, Tokyo, Japan). According to the method of Akbarbaglu et al. (2024) with minor modifications [6], three independent hydrolysis procedures were performed according to the different acid-base stabilities of amino acids. For the determination of 16 conventional amino acids (aspartic acid, threonine, serine, glutamic acid, proline, glycine, alanine, valine, isoleucine, leucine, tyrosine, phenylalanine, lysine, histidine, arginine, and methionine), approximately 0.1000 g of dried sample was accurately weighed into a hydrolysis tube, mixed with 10 mL of 6 mol/L hydrochloric acid and a few drops of phenol, purged with high-purity nitrogen for 30 s to eliminate oxygen, tightly sealed, and hydrolyzed at 110 °C for 22 h. For cystine detection, the sample was pre-oxidized with pre-cooled performic acid solution at 0 °C for 16 h, terminated with sodium metabisulfite, and then subjected to the same 6 mol/L HCl hydrolysis procedure; the cystine content was calculated according to the molar conversion of cysteic acid. For tryptophan determination, which is completely degraded under acidic conditions, the sample was hydrolyzed with 4 mol/L lithium hydroxide solution in a PTFE tube at 110 °C for 20 h under nitrogen protection, and the hydrolysate was neutralized to pH 4.0–5.0 after cooling. After hydrolysis, all cooled hydrolysates were quantitatively transferred to a 50 mL volumetric flask and diluted to the mark with ultrapure water. After filtration, 1 mL of filtrate was taken and evaporated to dryness with a nitrogen evaporator for derivatization. Subsequently, 100 μL of derivatization reagent (trimethylamine: acetonitrile: water = 1:2:2) and 100 μL of acetonitrile solution were added and mixed thoroughly, and the mixture was incubated for 1 h at room temperature in the dark. Afterwards, 1 mL of n-hexane was added for vortex extraction and static phase separation, and the lower aqueous phase was collected and filtered through a 0.22 μm microporous membrane. The obtained filtrate was determined by cation exchange chromatography coupled with post-column ninhydrin derivatization; gradient elution was performed with a sodium citrate buffer system, the derivatization temperature was set at 135 °C, and the detection wavelengths were 570 nm for conventional amino acids and 440 nm for proline. All samples were analyzed in triplicate, and the results were calculated on a dry basis using the external standard method.

### 2.4. Determination of Fatty Acid Composition

The analytical procedure was carried out according to GB 5009.168-2016 with minor modifications [28]. The fat extract was obtained using the method described in Section 2.2. Approximately 30–50 mg of the fat extract was transferred into a screw-cap glass test tube, and triglyceride of undecanoic acid (C11:0) was added as the internal standard. Four milliliters of 2 mol/L NaOH-methanol solution was added, and the mixture was sealed under a nitrogen atmosphere, then saponified in a 70 °C water bath for 30 min with intermittent shaking. After cooling to room temperature, 5 mL of 14% boron trifluoride-methanol (BF_3_-methanol) solution was added, and the mixture was kept at 70 °C for 5 min for methyl esterification. Five milliliters of n-hexane was added, and the mixture was vortex-shaken to extract fatty acid methyl esters. Saturated sodium chloride solution was added, and the mixture was allowed to stand for phase separation. The upper n-heptane layer was collected, dehydrated over anhydrous sodium sulfate, filtered through a 0.22 μm organic-phase membrane, and subjected to GC-FID analysis.

Fatty acids were quantified by gas chromatography using an internal-standard calibration method. An Agilent 7890A gas chromatograph (Agilent Technologies, Santa Clara, CA, USA) equipped with a flame-ionization detector (FID) and an SP-2560 capillary column (100 m × 0.25 mm × 0.2 μm, Supelco-Merck, Bellefonte, PA, USA) was used. High-purity nitrogen was employed as the carrier gas at a constant flow rate of 1.0 mL/min; split ratio, 100:1; injection volume, 1.0 μL. Injector temperature was set at 270 °C and detector temperature at 280 °C. The hydrogen flow was 40 mL/min, the air flow was 400 mL/min, and the make-up nitrogen flow was 25 mL/min. The oven temperature program was as follows: initial temperature of 100 °C, held for 13 min; increased to 180 °C at 10 °C/min, held for 6 min; increased to 200 °C at 1 °C/min, held for 20 min; and finally increased to 230 °C at 4 °C/min, held for 10.5 min. Individual fatty acids were identified by matching retention times against mixed fatty-acid methyl-ester reference standards. Method validation, including a spike-recovery test and repeatability, was performed; acceptable recoveries ranged from 80 % to 115 % (75–120% for minor fatty-acid components). The relative standard deviation (RSD) was used to evaluate repeatability (RSD ≤ 5% for major fatty acids and RSD ≥ 10% for minor fatty acids). The limit of detection (LOD, S/N = 3) and limit of quantitation (LOQ, S/N = 10) were determined based on the signal-to-noise ratio.

### 2.5. Extraction and Determination of Vitamin B_1_

According to GB 5009.84-2016 [29] with minor modifications, ground apricot kernel powder (2.0 g) was weighed, and approximately 50 mL of 0.1 mol/L hydrochloric acid was added. The mixture was mixed well and hydrolyzed in a boiling-water bath for 60 min. After cooling, the pH was adjusted to 4.0–4.5 with sodium acetate solution. The solution was quantitatively diluted to 100 mL and filtered. An aliquot of 2.0 mL of filtrate was transferred into a brown glass test-tube for pre-column derivatization under dim light conditions. Briefly, 1.0 mL of freshly prepared alkaline potassium ferricyanide solution was added to oxidize thiamine to fluorescent thiochrome. After vortex-mixing, 2.0 mL of n-butanol was added, vortex-shaken for 1.5 min, and kept static for phase separation. The upper n-butanol organic phase was collected, filtered through a 0.45 μm organic-phase filter membrane, and transferred to an amber HPLC vial for injection. Standard working solutions were subjected to the same derivatization procedure as the samples.

HPLC-fluorescence detection analysis was performed on an Agilent 1260 high-performance liquid chromatograph equipped with a fluorescence detector (FLD, Agilent Technologies, USA). Separation was achieved on a C18 reversed-phase column (250 mm × 4.6 mm, 5 μm; Agilent Technologies, USA). The column temperature was maintained at 30 °C. The mobile phase consisted of methanol and ammonium acetate buffer (10:90, *v*/*v*; 0.05 mol/L, pH 5.0). The flow rate was set at 1.0 mL/min, and total chromatographic run time was 18 min. Fluorescence detector parameters: excitation wavelength (Ex) = 245 nm; emission wavelength (Em) = 435 nm; and injection volume = 20 μL.

A thiamine hydrochloride standard (purity ≥ 99.0%, Sigma-Aldrich, St. Louis, MO, USA) was used for external-standard calibration. Stock standard solution (500 μg/mL) was prepared in 0.01 mol/L hydrochloric acid. A series of working calibration solutions were obtained by serial dilution, covering the linear range of 0.05–1.00 μg/mL. Acceptable recoveries for thiamine ranged from 80% to 115%; repeatability expressed as relative standard deviation (RSD) was required to be ≤10%. The limit of detection (LOD, S/N = 3) and limit of quantitation (LOQ, S/N = 10) were determined via signal-to-noise ratio.

### 2.6. Extraction and Determination of Vitamin E (α, γ, δ-Tocopherols)

The method was a slight modification of the method described by Hajam et al. (2025) [30]. Approximately 1.0 g of apricot powder was weighed into a saponification flask. Twenty milliliters of anhydrous ethanol, 2 mL of 10% ascorbic acid in ethanol, and 5 mL of 50% KOH were added. The flask was flushed with nitrogen for protection and refluxed in an 80 °C water bath for 30 min with continuous shaking. After cooling, water was added, and extraction was performed 3 times with n-hexane. The organic phases were combined and washed with water until neutral to remove alkali and water-soluble impurities. The organic phase was dried over anhydrous sodium sulfate, concentrated by rotary evaporation to near dryness, and diluted with methanol to a final volume of 10 mL. The solution was filtered through a 0.22 μm organic membrane filter and protected from light until analysis.

Analysis was carried out on an Agilent 1260 high-performance liquid chromatograph equipped with a fluorescence detector (FLD, Agilent Technologies, USA). A C18 reversed-phase column (250 mm × 4.6 mm, 5 μm, Agilent Technologies, USA) was used. The column temperature was maintained at 35 °C. Pure methanol served as the mobile phase at a flow rate of 1.0 mL/min. The injection volume was 20 μL. Fluorescence detector parameters were set to excitation wavelength (Ex) = 295 nm and emission wavelength (Em) = 330 nm. External-standard calibration curves were separately established using authentic standards of α-tocopherol, γ-tocopherol and δ-tocopherol (purity ≥ 98.0%, Sigma-Aldrich, USA).

The contents of α-, γ- and δ-tocopherol were expressed in mg/100 g (kernel dry-weight basis). Total vitamin E activity was calculated as α-tocopherol equivalents (α-TE, mg α-TE/100 g), using the formula α-TE = 1.0 × (α-tocopherol) + 0.1 × (γ-tocopherol + 0.01 × δ-tocopherol), where α-, γ- and δ-tocopherol were expressed in mg/100 g.

### 2.7. Determination of Mineral Element Content

The apricot kernel samples (0.5 ± 0.01) g were accurately weighed into polytetrafluoroethylene (PTFE) microwave-digestion vessels. Then, 5.0 mL of 70% concentrated nitric acid and 2.0 mL of hydrogen peroxide were added. Microwave digestion was performed using a microwave digestion instrument (MARS6, CEM Co., Matthews, NC, USA), referring to the method of Yan et al. (2025) [15]. After the digestion program was finished, the vessels were cooled down to room temperature in a fume hood. The digested solution was quantitatively transferred to a polypropylene centrifuge tube, and the residual acid was evaporated by heating to near dryness to remove nitrogen-oxide fumes. Subsequently, the residue was dissolved with a small amount of 2% nitric acid solution, and the solution was quantitatively made up to 25.0 mL with ultrapure deionized water. A reagent blank was prepared following exactly the same digestion and dilution procedure without sample material.

Determinations of K, Ca and Zn were performed by inductively coupled plasma-mass spectrometry (ICP-MS, Agilent 7500, Agilent Technologies, USA). The ICP-MS operating parameters were set as follows: radio-frequency power, 1550 W; carrier gas (argon) flow rate, 1.05 L/min; make-up gas flow rate, 0.15 L/min; spray-chamber temperature, 2 °C; and sampling depth, 8 mm. External-standard calibration was adopted for quantification, and standard-series solutions were prepared using a 2% nitric acid matrix-matching solution. Method quality-control measures included reagent blanks, duplicate sample parallel tests, spike-recovery experiments, and determination of limit of detection (LOD, S/N = 3) and limit of quantitation (LOQ, S/N = 10). The acceptable spike-recovery range for mineral elements was 85–110%, and the relative standard deviation (RSD) for sample duplicates was required to be ≤10%. The contents of K, Ca and Zn were expressed as mg/kg on a dry-weight basis.

### 2.8. Data Processing and Statistical Analysis

Each sample was collected with three biological replicates, and each biological replicate contained three technical replicates. All physicochemical and nutritional indicator measurements were performed in triplicate.

Excel 2023 was used to calculate the minimum, maximum, mean, standard deviation, coefficient of variation, and Shannon-Wiener genetic diversity index (*H*′) for each nutritional parameter. Z-score-normalized data were subjected to Pearson correlation analysis and principal component analysis (PCA) using SPSS 24.0. The significance level for correlation analysis was set at *p* < 0.05. Origin 2021 was employed for cluster analysis, frequency distribution analysis, and graphing. Hierarchical cluster analysis (HCA) was performed to assess the similarity among samples. The unweighted pair-group method using arithmetic averages (UPGMA, average-linkage) was adopted as the clustering linkage rule, and Euclidean distance was used to construct the dendrogram.

The relevant indicators were calculated as follows:

Z-score standardization was performed using the following formula: Z_ij_ = (X_ij_ − $\bar{X}$_j_)/S_j_ (j = 1, 2, 3, …, n), where X_ij_ represents the original value of the j-th indicator for the i-th cultivar, $\bar{X}$_j_ is the mean of the j-th indicator across all cultivars, and S_j_ is the corresponding standard deviation.

Genetic diversity index Shannon–Wiener (*H*′) = −ΣP_i_ × log_2_P_i_, where P_i_ is defined as the percentage of the number of accessions in the i-th grade for a given trait relative to the total number of accessions. For each component, it was divided into five levels according to the content.

Membership function value was calculated using the formula μ(X_ij_) = (X_ij_ − X_min_)/(X_max_ − X_min_) (j = 1, 2, 3, …, n), where μ(X_ij_) is the membership function value of the j-th indicator for the tested sample, X_ij_ is the value of the j-th nutritional indicator for the i-th cultivar, X_min_ is the minimum value of the j-th indicator, and X_max_ is the maximum value of the j-th indicator.

Principal component score (F_ij_) was calculated using the formula F_ij_ = Σ[μ(X_ij_) × R_j_] (j = 1, 2, 3, …, n), where R_j_ is the principal component score for the j-th indicator.

Comprehensive evaluation value (D) was calculated using the formula D = Σ(F_ij_ × W_j_) (j = 1, 2, 3, …, n), where W_j_ is the proportion of the variance contribution rate of the j-th principal component, i.e., the weight.

## 3. Results

### 3.1. Frequency Distribution and Diversity Index of Nutritional Components in Kernels from 141 Apricot Cultivars

#### 3.1.1. Nutritional Diversity Among Cultivars Based on Differences in Kernel Nutritional Components

A total of 12 fatty acids were detected in the kernel samples tested in this study, with total content ranging from 19.0 to 49.7 g/100 g. Among these, five fatty acids had contents exceeding 0.1 g/100 g: oleic acid (7.91–35.2 g/100 g), linoleic acid (8.4–17.1 g/100 g), palmitic acid (0.989–2.61 g/100 g), stearic acid (0.148–0.7 g/100 g), and palmitoleic acid (0.121–0.424 g/100 g). During the evaluation process, four indicators with relatively high contents were selected: total fatty acids, oleic acid, linoleic acid, and palmitic acid. Additionally, 18 amino acids were detected in the kernels, including eight essential amino acids for the human body (with content ranging from 5.37 to 9.67 g/100 g). The total content of these 18 amino acids was used as an evaluation indicator in this study.

As shown in Table 2, the genetic diversity index (*H*′) for the 15 nutritional components among the kernel quality indicators of the 141 apricot resources ranged from 1.52 to 2.09, all exceeding 1.0. This indicates significant variation in nutrition levels among different cultivars and rich diversity in kernel quality. Notably, fat, protein, oleic acid, linoleic acid, and vitamin E exhibited the highest genetic diversity, with H’ values all above 2.0. The coefficients of variation for these nutritional indicators among cultivars ranged from 8.49% to 35.43%. Protein content showed the smallest variation among cultivars, with the lowest coefficient of variation at only 8.49%. The contents of oleic acid, the mineral element potassium, and vitamins (including vitamin B_1_, α-tocopherol, and δ-tocopherol) varied considerably among varieties, with coefficients of variation of 25.78%, 31.55%, 26.59%, 35.43%, and 34.73%, respectively. The range values were highest for zinc, fat, fatty acids, and oleic acid. Both the coefficient of variation and the range for oleic acid were relatively large, indicating substantial variation in oleic acid content among different kernel varieties.

#### 3.1.2. Frequency Distribution of Nutritional Component Contents in Kernels

**Figure 1:**
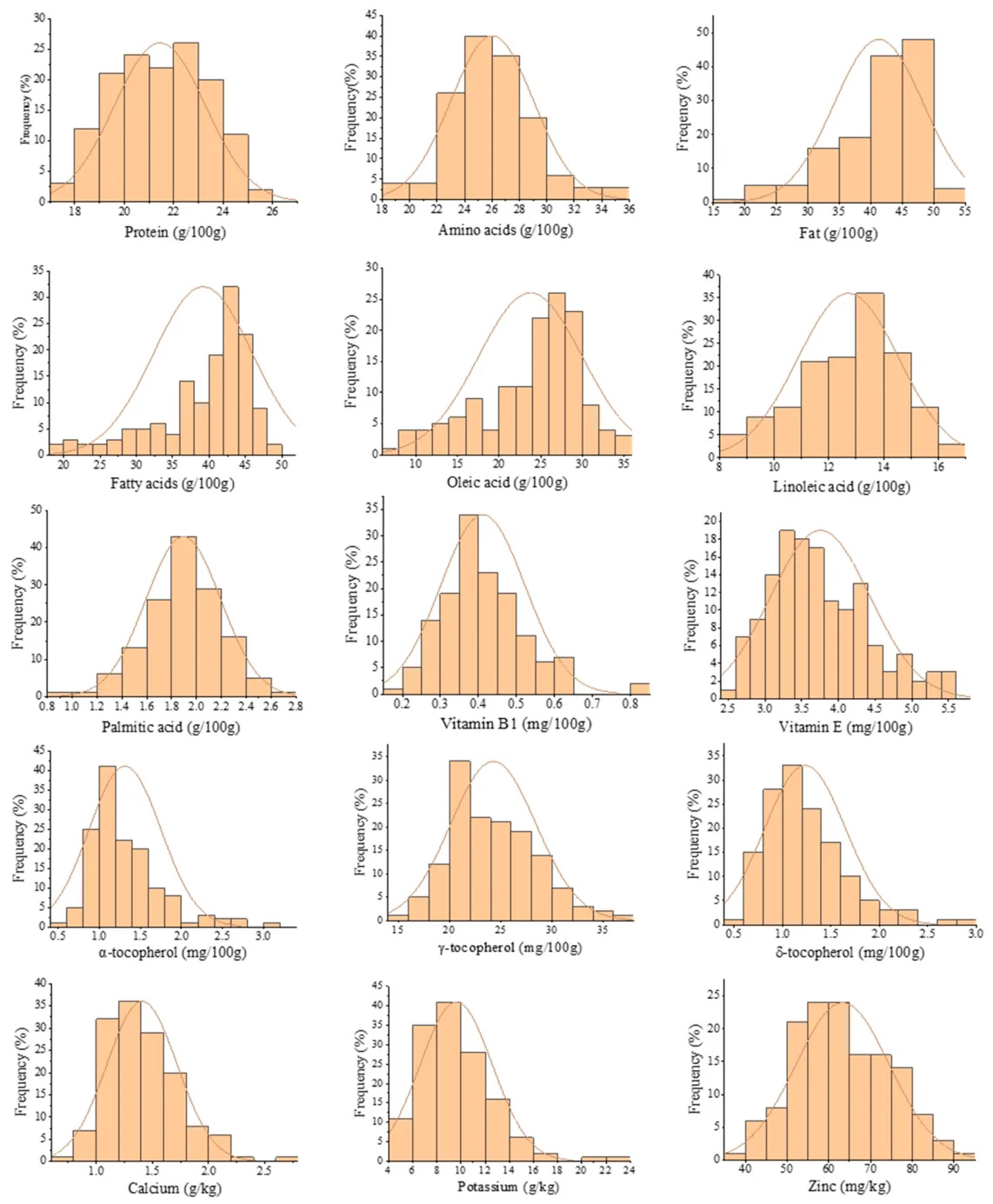
Frequency distribution histograms of 15 nutritional components in kernels of 141 apricot cultivars.

The frequency distribution histograms of 15 nutritional components in kernels (Figure 1) showed that each component exhibited a normal or approximately normal distribution. In terms of frequency distribution by class, the protein content had the highest frequency in the range of 22.0–23.1 g/100 g, accounting for 22.7%; amino acids had the highest frequency in the range of 24–28 g/100 g, accounting for 55.3%; fat content had the highest frequency in the range of 40–50 g/100 g, accounting for 70.2%; fatty acid content had the highest frequency in the range of 40–45 g/100 g, accounting for 51.1%; the frequency distributions of palmitic acid, oleic acid, and linoleic acid were concentrated in the ranges of 1.8–2.0 g/100 g, 25–30 g/100 g, and 13–14 g/100 g, accounting for 30.5%, 51.1%, and 27.7%, respectively; the highest frequency distributions of vitamin B_1_, vitamin E, γ-tocopherol, α-tocopherol, and δ-tocopherol were concentrated in the ranges of 0.35–0.4 mg/100 g, 3.0–3.8 mg/100 g, 20–22 mg/100 g, 1.0–1.2 mg/100 g, and 0.85–1.4 mg/100 g, accounting for 24.1%, 48.2%, 24.1%, 33.3%, and 60.3%, respectively; the highest frequency distributions of the mineral elements zinc, potassium, and calcium were concentrated in the ranges of 50–65 mg/kg, 6.5–10 g/kg, and 1–1.5 g/kg. Overall, the frequency distributions of different nutritional components were wide, indicating significant differences in kernel quality among the 141 apricot accessions.

### 3.2. Correlation Analysis of the 15 Nutritional Quality Indicators

**Figure 2:**
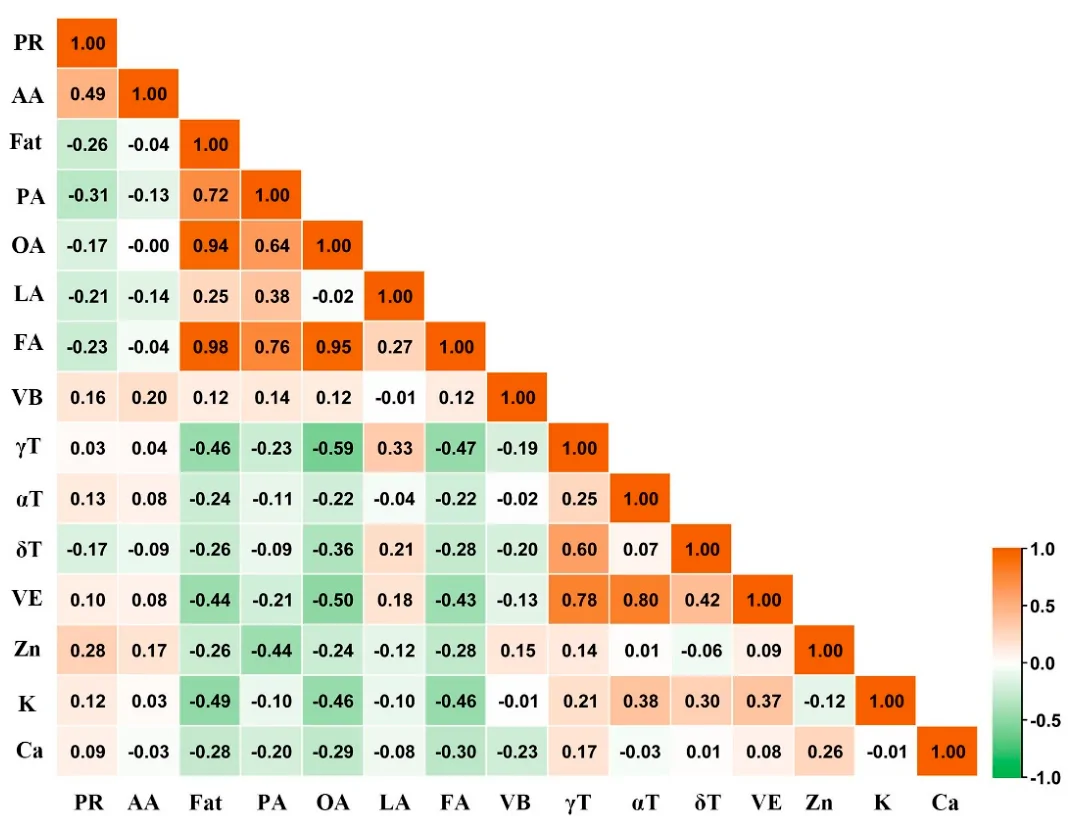
Pearson correlation heatmap of 15 nutritional components in apricot kernels of 141 germplasm accessions. The color gradient represents the Pearson correlation coefficient ranging from −1.0 (green) to 1.0 (orange), *p* < 0.05. PR, protein; AA, total amino acids; Fat, crude fat; PA, palmitic acid; OA, oleic acid; LA, linoleic acid; FA, total fatty acids; VB1, thiamine (vitamin B_1_); γT, γ-tocopherol; αT, α-tocopherol; δT, δ-tocopherol; VE, vitamin E; Zn, zinc; K, potassium; Ca, calcium.

Pearson correlation analysis was used to assess linear relationships between nutritional indices. The significance level for correlation analysis was set at *p* < 0.05, and the correlation coefficient was denoted as r. As shown in Figure 2, red in the legend indicates a positive correlation, with values closer to 1 representing a stronger positive correlation; conversely, blue indicates a negative correlation, with values closer to -1 representing a stronger negative correlation. Fatty acid (FA) content shows extremely significant pairwise positive correlations with oleic acid (OA), palmitic acid (PA), and fat content, with Pearson correlation coefficients all exceeding 0.717. The highest correlation is between fatty acids and fat, with a coefficient of 0.981. Vitamin E exhibits extremely significant positive correlations with α-tocopherol and γ-tocopherol, with correlation coefficients of 0.801 and 0.781, respectively. Oleic acid shows significant pairwise negative correlations with vitamin E and γ-tocopherol, with correlation coefficients of −0.503 and −0.588, respectively. It is evident that the different nutritional components of kernels are interconnected and influence each other, and the correlations among these components can be better understood from the perspective of genetic mechanisms.

### 3.3. Principal Component Analysis of Kernel Nutritional Quality Indicators

Factor analysis was conducted on 15 nutritional quality indicators of kernels. The results showed that the KMO value was 0.5 and the probability value of Bartlett’s test of sphericity was 0.000 (*p* < 0.001), indicating that the data were correlated and suitable for principal component analysis.

To clarify the contribution of different nutritional components to kernel quality diversity and to simplify the evaluation indicators, principal component analysis (PCA) was performed on 15 nutritional quality indicators of 141 apricot kernel accessions. Data were Z-score standardized prior to analysis based on a correlation matrix. Principal components were extracted with eigenvalues greater than 1, and Varimax orthogonal rotation was applied to enhance interpretability. This procedure yielded five principal components explaining a cumulative total of 75.614% of the total variance (Table 3), suggesting that these five PCs adequately represent most of the genetic information conveyed by the original 15 indicators.

As shown in Table 3, the first principal component (PC1) had an eigenvalue of 4.975 and a contribution rate of 33.167%, with the highest eigenvector loadings for fatty acids, oleic acid, and fat content, primarily reflecting the oil content of kernels. The second principal component (PC2) had an eigenvalue of 2.352 and a contribution rate of 15.68%, with the highest loading for linoleic acid, mainly reflecting the unsaturated fatty acid content. The third principal component (PC3) had an eigenvalue of 1.666 and a contribution rate of 11.108%, with the highest loadings for α-tocopherol and amino acids, primarily representing fat-soluble vitamins and amino acid content. The fourth principal component (PC4) had an eigenvalue of 1.31 and a contribution rate of 8.732%, with the highest loadings for zinc and potassium, reflecting trace element content. The fifth principal component (PC5) had an eigenvalue of 1.039 and a contribution rate of 6.926%, with the highest loading for vitamin B, representing water-soluble vitamin B content. Based on the results of PCA and correlation analysis, the 15 nutritional indicators were simplified into seven core indicators: fatty acids, oleic acid, linoleic acid, amino acids, zinc, α-tocopherol, and vitamin B_1_. Seven core indicators were screened from the initial 15 variables based on a combined strategy of principal component loadings, redundancy reduction and nutritional significance. Variables with high loadings on distinct principal components were preferentially retained. Highly correlated redundant variables were eliminated to reduce information overlap. Meanwhile, nutritional importance was considered to ensure that the selected indicators covered multiple nutrient categories, including lipids, amino acids, minerals and vitamins, to comprehensively reflect the nutritional characteristics of apricot kernels. These core indicators serve as an important basis for evaluating kernel nutritional quality and for germplasm resource selection.

**Figure 3:**
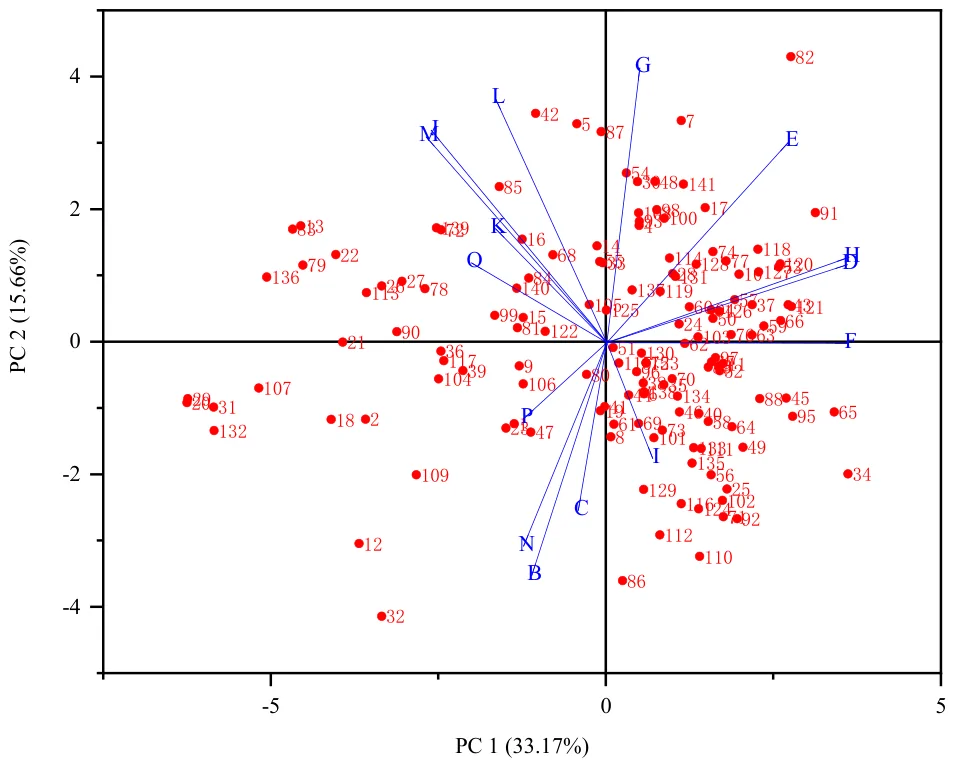
Bi-plot of principal component analysis based on 15 nutritional components for 141 apricot accessions. Red dots represent individual cultivars, and blue vectors indicate nutritional variables (A–O, representing PR-Ca). The percentages in parentheses represent the variance explained by each principal component. The distance between dots reflects the similarity of nutritional profiles among cultivars. Vector length indicates the contribution of each nutritional variable to the two-dimensional plane; the angle between vectors reflects the correlation between variables.

A bi-plot was generated based on PC1 and PC2 to visualize germplasm differentiation and variable relationships (Figure 3). Germplasm accessions located close to each other on the bi-plot exhibited similar nutritional compositions, and several distinct groups could be observed. Some individual accessions (e.g., No.82 ‘Kuhehonglian’, No.86 ‘Liguangxing’) were clearly separated from the main cluster, representing unique nutritional profiles. The blue vectors in the bi-plot illustrate the contribution and correlation of each nutritional variable; variables with small angles between vectors were positively correlated, whereas vectors pointing in opposite directions indicated negative correlation. These findings provide valuable references for kernel quality evaluation and the selection of elite germplasm. It should be noted that PC1 and PC2 together explained only 48.83% of the total variation (33.17% and 15.66%, respectively), indicating that the nutritional variation among the tested germplasm was complex and that substantial information resided in the third and subsequent principal components (Table 3). Therefore, hierarchical cluster analysis was employed as complementary evidence for germplasm classification.

### 3.4. Cluster Analysis of Apricot Germplasm Resources

**Figure 4:**
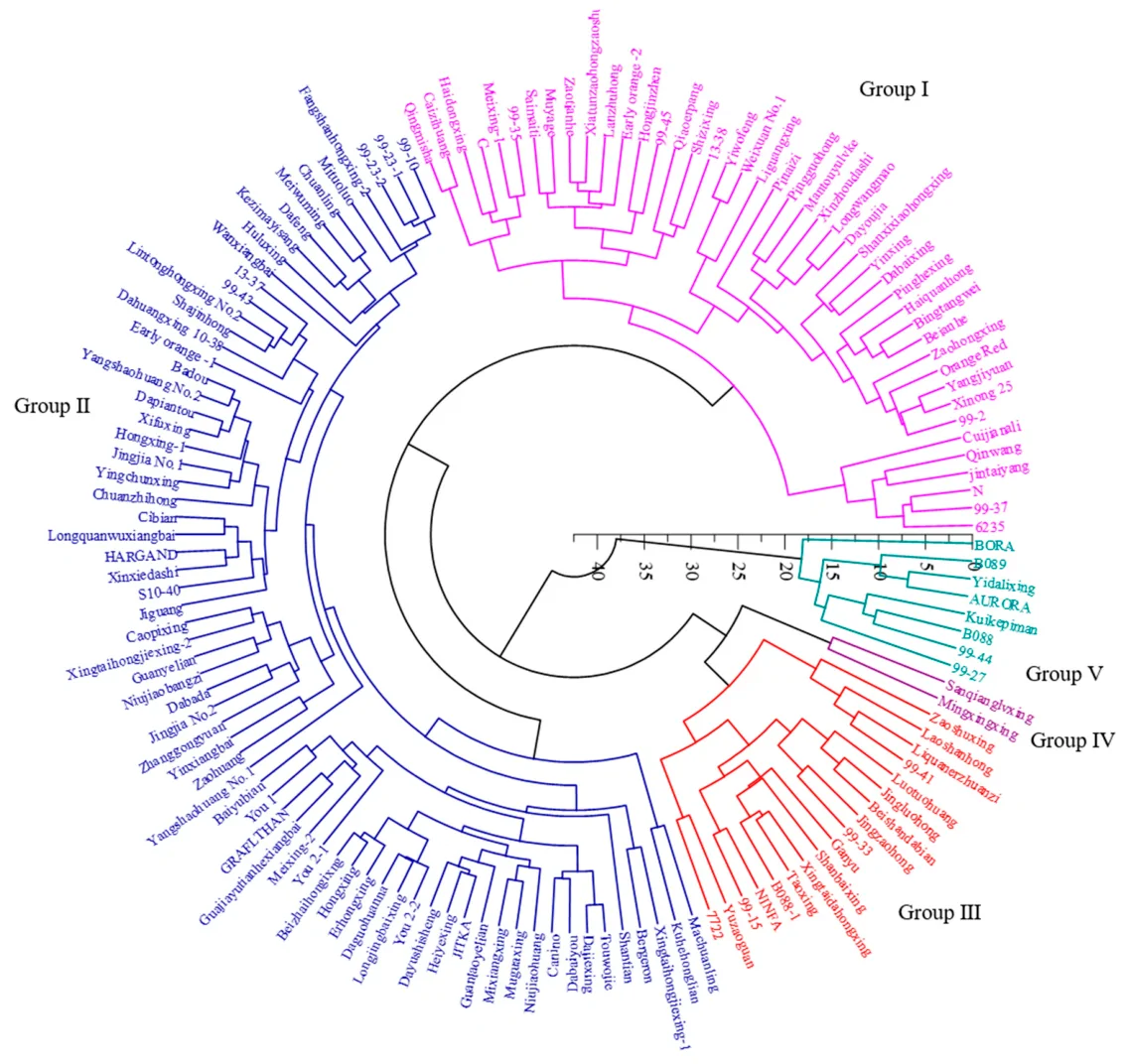
Circular clustering plot of 141 apricot cultivars based on nutritional quality traits. The circular plot shows the grouping results of cultivars by hierarchical cluster analysis (UPGMA, Euclidean distance). Each branch represents an individual apricot cultivar, and the color segments in the outer ring indicate the cluster groups classified according to nutritional phenotypic variation.

A cluster analysis was conducted based on 15 nutritional characteristics of kernels from 141 apricot cultivars to investigate the genetic relationships among them. At a Euclidean distance of 19.5, the tested apricot accessions were divided into five groups (Figure 4), and the main nutritional indicators of the germplasm in each group were statistically analyzed (Table 4).

Group I consisted of 44 apricot germplasm resources, accounting for 31.21% of the total. The kernels in this group exhibited relatively high contents of fat, oleic acid, fatty acids, and zinc, with mean values of 42.21 g/100 g, 24.15 g/100 g, 39.93 g/100 g, and 72.84 mg/kg, respectively. The coefficients of variation were relatively low at 9.90%, 17.16%, 9.73%, and 9.63%, respectively. The potassium content was the lowest among all groups, but the coefficient of variation was relatively high (26.85%). Among the cultivars, ‘Longwangmao’ kernel had the highest content of oleic acid, an unsaturated fatty acid, in this group, reaching 31.3 g/100 g, with relatively high protein and amino acid contents as well. The apricot kernels in this group generally had high comprehensive nutritional value.

Group II included 70 apricot accessions, accounting for 49.65% of the total. This group had the highest contents of fat, oleic acid, and fatty acids among the five groups, with mean values of 45.13 g/100 g, 27.34 g/100 g, and 42.97 g/100 g, respectively. These cultivars can be used as parents for breeding kernel-using apricot varieties with high oil content. Notably, seven cultivars including ‘Machuanling’, ‘Kuhehonglian’, ‘GRAFLTHAN’, ‘Gujiayu Tianhexiangbai’, ‘Guanlaoyelian’, ‘Xingtai Hongjiexing-1’, and ‘Chuanzhihong’ exhibited the highest fat and total fatty acid contents within this group, with fat content ranging from 48.09 to 51.85 g/100 g and fatty acid content ranging from 46.5 to 49.7 g/100 g. Three traditional kernel-using apricot cultivars, ‘You 1’, ‘Baiyubian’, and ‘You 2-1’, which ranked fourth, fifth, and sixth in oleic acid content within this group, can be used as raw materials for the processing of functional foods rich in highly unsaturated fatty acids.

Group III comprised 17 apricot accessions, accounting for approximately 12.06% of the total. The vitamin content of the kernels of this group was relatively high, particularly γ-tocopherol, which had the highest mean value among the five groups at 28.97 mg/kg, with a moderate coefficient of variation of 15.49%. Five cultivars, namely ‘Jingzaohong’, ‘Yuzaoguan’, ‘99-33’, ‘Ganyu’, and ‘Jingluohong’, ranked in the top five for γ-tocopherol content within this group, with contents ranging from 31.26 to 37.7 mg/100 g. These cultivars are suitable for processing functional foods with high vitamin content.

Group IV had the fewest accessions, containing only two cultivars, ‘Mingxingxing’ and ‘Sanqianglvxing’, accounting for 1.42% of the total. This group exhibited relatively higher protein, amino acid, α-tocopherol, vitamin E, and potassium contents than the other four groups. These two cultivars may be potential candidates either as direct sources for high-protein foods or as raw materials for food processing.

Group V consisted of eight apricot cultivars, accounting for approximately 5.67% of the total. The apricot kernels in this group had significantly higher contents of the mineral element zinc and calcium compared to resources in other groups, with mean values of 82.53 mg/kg and 1.88 g/kg, respectively. The coefficient of variation for zinc content was low (6.5%), while that for calcium content was relatively high (26.44%). The oleic acid content (10.53 g/100 g) was significantly lower than in the other four groups. The resource ‘99-44’ from the Czech Republic had the highest calcium content at 2.68 g/kg. These cultivars could serve as potential sources for functional foods characterized by high calcium and low oleic acid content. Additionally, in terms of variety origin, apart from ‘Kuikepiman’, which belongs to the Xinjiang variety group, the other seven resources in this group are from European variety groups abroad.

The five germplasm groups identified above can be comprehensively analyzed and selected in practical breeding work based on different breeding objectives.

### 3.5. Comprehensive Evaluation of Kernel Nutritional Quality Indicators

The nutritional quality indicators of kernels were reduced to five comprehensive factor indices. The comprehensive D value was calculated using the membership function method combined with the weights derived from principal component analysis. First, the membership function values for different materials and indicators were calculated based on the membership function formula. Then, the principal component scores F for different materials and nutritional indicators were obtained using the principal component analysis results and membership function values. Finally, the comprehensive evaluation D value was calculated based on the variance contribution rate of each principal component as the weight. A higher D value indicates better comprehensive nutritional quality for the 15 nutritional components of the material. The germplasm materials were ranked by D value, and the top 20 were selected, as shown in Table 5. F1–F5 represent the scores of the five principal components for kernel nutritional quality, and D represents the comprehensive evaluation score.

The apricot cultivar ‘Kuhehonglian’ exhibited the highest nutritional content, with a comprehensive D value (1.68) surpassing that of other varieties. Its F1 score (2.15) and F2 score (2.10) were relatively high, indicating the superiority of ‘Kuhehonglian’ kernels in key quality traits such as fatty acid and vitamin content. The D values of ‘Machuanling’, ‘Guajiayutianhexiangbai’, ‘Guanlaoyelian’, and ‘GRAFLTHAN’ ranked second to fifth, respectively. These four cultivars had high F1 scores, demonstrating significant advantages in nutritional quality traits such as oleic acid, fatty acids, and palmitic acid.

The cultivars ‘Chuanzhihong’, JITKA, 99-23-1, ‘Yangshaohuang No.1’, and ‘Mantouyulvke’ exhibited high F3 scores, indicating their superiority in nutritional quality traits such as protein and amino acids. ‘Guanlaoyelian’ and ‘Mantouyulvke’ had the highest F4 scores, reflecting their high quality in trace elements such as zinc and potassium. The two apricot cultivars ‘Dayushisheng’ and ‘Mantouyulvke’ had the highest F5 scores, indicating their superior quality in terms of vitamin B content.

## 4. Discussion

### 4.1. High Nutritional Variety Among Different Apricot Cultivars

Almond (*Prunus dulci*) kernels are widely recognized and favored as a high-nutrient nut variety due to their richness in unsaturated fatty acids and high-quality protein [30,31]. Cao et al. performed a comparative analysis of kernel proteins from apricot (*Prunus armeniaca* Lam.) and long-stalked almond (*Amygdalus pedunculata* Pall.), providing structural, functional, and digestive insights for sustainable food applications [32]. Almonds (Prunus dulcis) contain 18–25% protein (dry weight) [33]. Similar to this, the crude fat and protein contents in 44 varieties of Xinjiang almonds ranged from 38.18% to 47.81% and from 18.80 to 24.78%, respectively [34]. In this study, it was found that the nutritional components of apricot kernels are extremely close to those of almond kernels. Notably, the fat content in some apricot kernel varieties is significantly lower than that in almond kernels, while the maximum total amino acid and essential amino acid contents in certain cultivars are higher than those in almond kernels. This indicates that the nutritional variation of apricot kernels is greater than that of almond kernels. The protein content in apricot kernels ranged from 17.50 to 25.97 g/100 g, fat content from 16.64 to 51.85 g/100 g, total amino acid content from 18.66 to 35.15 g/100 g, and essential amino acid content from 5.37 to 9.67 g/100 g (Table S1). The protein and total amino acid contents in the 141 apricot kernels were higher than those of European apricot varieties. For the 15 European apricot varieties, the total amino acid content of the kernels ranged from 125.70 to 305.32 mg·g^−1^ of dried kernel [35]. Among 32 apricot cultivars, the protein content of kernels ranged from 14.56% to 28.77%. Moreover, Hybrid LI-3-6, originating from China, showed a high protein content [36]. The fatty acid composition was reasonably balanced. In almond kernels, the ratio of total unsaturated fatty acids (USFA) to total saturated fatty acids (SFA) is approximately 14:1 [37]. In this study, the USFA-to-SFA ratio in apricot kernels was 15.5:1, with a higher USFA level (Table S2). It is evident that apricot kernels not only contain protein and fatty acids comparable to almond kernels but also exhibit a wider range of variation among cultivars, laying a material foundation for developing high-protein, low-fat products.

The coefficient of variation and Shannon-Wiener diversity index of phenotypic traits can reflect the degree of individual differences and the level of biodiversity [20]. In this study, the coefficients of variation for various parameters ranged widely, from 8.49% to 35.43%. This range is higher than the coefficient of variation (8–10.4%) for 22 quantitative traits in Prunus mandshurica [38] and consistent with the coefficient of variation (8.38–31.68%) for 16 quantitative traits related to leaves and kernels in wild apricots from the Beijing region [39]. Typically, a coefficient of variation exceeding 10% indicates substantial individual differences [40,41]. Among the 15 nutritional indicators in this study, 14 had coefficients of variation greater than 10%, and 12 of these exceeded 15%, reflecting high genetic variation. However, the coefficient of variation for protein content was the smallest (8.49%), lower than that for bitter apricot kernels in the Northwest region (13.47%), while the coefficient of variation for fat content (19.21%) was higher than that for bitter apricot kernels in the Northwest region (7.49%) [42] and also higher than that for Siberian apricot kernels (13.30%) [26]. This discrepancy may be attributed to substantial differences in sample sizes and significant variations in the sampling regions covered by the studies. It may also be that kernel nutrition varies significantly among different apricot species: *Prunus armeniaca*, *Prunus mandshurica* and *Prunus Siberia*.

The diversity index for nutritional components in apricot kernels among different cultivars in this study was also higher than that for previously studied phenotypic traits in apricot germplasm, such as leaf, fruit, kernel, and seed morphology [38,39,43]. The rich variation observed in this study provides possibilities for discovering new germplasm and breeding superior kernel-use apricot varieties to satisfy different nutritional requirements.

### 4.2. Principal Component Analysis Is an Effective Method for the Scientific Evaluation of Extensive Apricot Germplasm

Previous studies applied PCA to fruit quality traits for germplasm phenotypic assessment. For example, 45 pomegranate cultivars were grouped into four clusters based on 18 fruit quality indicators, and elite accessions were screened using comprehensive evaluation scores [18]. Zhang et al. evaluated phenotypic variation of pear (*Pyrus* L.) germplasm using fruit traits; 17 out of 39 traits were selected via PCA and stepwise regression, accounting for 99.3% of total variation and serving as indicators to evaluate 570 pear accessions [20]. For Castanea mollissima, PCA extracted four principal components explaining 76.23% of total variance from 23 traits of 32 superior plants, and accessions with high comprehensive quality were identified to support germplasm utilization [44]. In the present study, five principal components were extracted from the 15 nutritional traits of apricot kernels, with a cumulative contribution rate of 75.614%, capturing the primary variation in kernel nutritional phenotypes. Variables with high absolute loading values in each principal component were recognized as major contributors to the phenotypic differences in kernel nutritional composition. Combined with correlation analysis, seven core indicators were screened from the 15 nutritional components to simplify the evaluation system. This set of indicators reduces the complexity of multi-index assessments. The findings offer phenotypic data and a simplified evaluation framework for exploring apricot kernel nutritional characteristics and may provide a reference for further utilization of apricot germplasm resources.

### 4.3. Exploration and Innovative Utilization of High-Nutrition Kernel Germplasm

Germplasm serves as the foundation for variety improvement and genetic research. Over the past decade, with advancements in molecular biology technologies, a significant reduction in genome sequencing costs, and rapid progress in bioinformatics, the regulatory genes and pathways governing many important phenotypic traits in fruit trees, such as flesh color in apples and cherries, stone cells in pears, and fruit shape in flat peaches, have been elucidated using specific variety resources as research materials [45,46,47,48]. This has greatly advanced targeted breeding efforts in fruit trees and laid the groundwork for the development of specialty fruit products.

Apricot is a type of fruit tree primarily cultivated for its flesh and kernels. Both the fruit and kernels possess high edible and medicinal value [12,49], making them highly sought-after premium commodities in domestic and international markets. Kernels can be processed into various products, including kernel oil, crude kernel protein powder, high-fiber dried apricots, crispy kernels, split apricot kernels, kernel beverages, kernel pudding, kernel paste, kernel pastries, and kernel pickles [5,50,51,52]. Apricot trees are widely cultivated worldwide, with a rich diversity of varieties. Previous studies have utilized molecular approaches to investigate the origin and classification of apricot accessions [53,54], while others have assessed certain nutritional components of kernels in a limited number of varieties [14,23,55]. However, comprehensive evaluations of multiple nutritional indicators across a large number of cultivars remain scarce. This study simultaneously measured 15 quality indicators of kernels from 141 apricot cultivars, including protein content, various amino acid components, fatty acid components, fat content, vitamin components, and mineral elements. This provides new data and a theoretical basis for a comprehensive understanding of the diversity or specificity of kernel nutritional quality in major apricot cultivars, laying the foundation for the processing and utilization of specialty cultivars. Based on comprehensive evaluation scores, the nutritional quality of kernels from the 141 apricot cultivars was ranked, and 11 cultivars with distinctive nutritional value were selected: ‘Kuhehonglian’, ‘Machuanling’, ‘Guajiayutianhexiangbai’, ‘Guanlaoyelian’, ‘GRAFLTHAN’, ‘Chuanzhihong’, JITKA, 99-23-1, ‘Yangshaohuang-1’, ‘Mantouyulvke’, and ‘Dayushisheng’. Through cluster analysis, superior parental lines were preliminarily screened out for breeding kernel varieties satisfying specific nutritional requirements, such as high unsaturated fatty acid (e.g., oleic acid) content, high vitamin content, high protein content, and high calcium content with low oil content. Clarifying the nutritional characteristics of kernels from different apricot germplasm materials laid a theoretical foundation for improving the processing and utilization of apricot kernels. The findings also provide references for the breeding of high-quality kernel-used cultivars.

Meanwhile, it should be noted that apricot kernels may contain cyanogenic glycosides, mainly amygdalin [56], which may produce cyanide and pose potential food safety risks. This study only characterized the nutritional components of apricot kernels and did not perform a food safety evaluation. The nutritional ranking and comprehensive score only reflect phenotypic nutritional characteristics and do not indicate suitability for direct human consumption. For food applications such as functional foods, beverages, pastries and apricot kernel paste, cultivar selection should consider both nutritional quality and amygdalin-related cyanogenic potential. Suitable processing treatments are necessary to reduce cyanogenic glycosides before edible utilization.

### 4.4. Study Limitations

This study has several limitations that should be noted. First, all samples were harvested from one site in a single year; environmental factors and inter-annual variation may alter the nutritional profiles of apricot kernels. Second, only phenotypic nutritional traits were investigated, and no molecular genetic data were obtained to characterize the germplasm. Third, cyanogenic glycosides (amygdalin), a critical safety indicator, were not quantified in the present work. The comprehensive evaluation score and cultivar ranking are strongly dependent on the selected variables and weighting scheme. Fourth, lipid-related variables were relatively overrepresented in the model. Finally, these phenotypic results were obtained from a single site and single year trial. Multi-location and multi-year tests are required to verify trait stability before using these germplasms in breeding programs. The selected germplasms are regarded as candidate materials for further comprehensive evaluation rather than proven superior parents.

## 5. Conclusions

In this study, 15 nutritional components in apricot kernels from 141 cultivars were determined. Significant differences in kernel nutrition were observed among different cultivars. Five principal components were extracted through principal component analysis, with a cumulative contribution rate of 75.614%, effectively capturing the essential information of the 15 nutritional indicators. In combination with correlation analysis, key components affecting the comprehensive nutritional quality of apricot kernels were identified, namely oleic acid, linoleic acid, total fatty acids, amino acids, α-tocopherol, vitamin B_1_, and zinc. Further cluster analysis divided the 141 apricot accessions into five groups at a Euclidean distance of 19.5, and cultivars with outstanding traits within each group were identified. Based on the comprehensive scores calculated using the membership function and principal component scores, the 141 accessions were ranked according to their overall nutritional quality, and the top 20 cultivars with excellent comprehensive nutritional quality were selected. These findings provide a theoretical basis and material support for the efficient development and utilization of apricot germplasm and promote the development of the apricot industry in functional kernel products.

## Figures and Tables

**Table 1:** Information on the tested apricot accessions in this study.

No.	Cultivar	No.	Cultivar	No.	Cultivar
1	6235	48	Dabada	95	Meixing-2
2	7722	49	Dabaixing	96	Meiwuming
3	99-2	50	Dabaiyou	97	Mituoluo
4	99-10	51	Dafeng	98	Muguaxing
5	Qingmisha	52	Daguohuanna	99	Muyage
6	S10-40	53	Dapiantou	100	Niujiaohuang
7	13-37	54	Dahuangxing 10-38	101	Pinaizi
8	13-38	55	Dajiexing	102	Pingguohong
9	99-15	56	Baiyubian	103	Pinghexing
10	99-23-1	57	JITKA	104	Qinwang
11	99-23-2	58	Dayoujia	105	Qiaoerpang
12	99-27	59	Dayushisheng	106	Saimaiti
13	99-33	60	Early orange -1	107	Sanqianglvxing
14	99-35	61	Early orange -2	108	Shajinhong
15	99-37	62	Canino	109	Shanbaixing
16	99-41	63	Bergeron	110	Shantian
17	99-43	64	Fangshanhongxing-2	111	Shanxixiaohongxing
18	99-44	65	Guajiayutianhebai	112	Shizixing
19	99-45	66	Guanlaoyelian	113	Taoxing
20	AURORA	67	Guanyelian	114	Touwojie
21	B088	68	Haidongxing	115	Wanxiangbai
22	B088-1	69	Haiquanhong	116	Weixuan No.1
23	NINFA	70	Heiyexing	117	Xingtaidahongxing
24	Orange Red	71	Jiguang	118	Xifuxing
25	Erhongxing	72	Hongjinzhen	119	Xinong 25
26	Ganyu	73	Hongxing	120	Xingtaihongjiexing-1
27	Jintaiyang	74	Hongxing-1	121	Xingtaihongjiexing-2
28	Mingxingxing	75	Huluxing	122	Xiatunzaohongzaoshu
29	Mixiangxing	76	Jingjia No.1	123	Xinxiedashi
30	Niujiaobangzi	77	Jingjia No.2	124	Xinzhoudashi
31	B089	78	Jingluohong	125	Yangjiyuan
32	BORA	79	Jingzaohong	126	Yangshaohuang No.1
33	C	80	Kezimayisang	127	Yangshaohuang No.2
34	GRAFLTHAN	81	Lanzhuhong	128	Yingchunxing
35	HARGAND	82	Kuhehonglian	129	Yiwofeng
36	N	83	Kuikepiman	130	Yinxiangbai
37	Badou	84	Laoshanhong	131	Yinxing
38	Beianhe	85	Liquanerzhuanzi	132	Yidalixing
39	Beishandabian	86	Liguangxign	133	You 1
40	Beizhaihongixng	87	Lintonghongxing No.2	134	You 2-1
41	Bingtangwei	88	Longjingbaixing	135	You 2-2
42	Caizihuang	89	Longquanwuxiangbai	136	Yuzaoguan
43	Caopixing	90	Luotuohuang	137	Zaohongxing
44	Chuanling	91	Machuanling	138	Zaohuang
45	Chuanzhihong	92	Longwangmao	139	Zaoshuxing
46	Cibian	93	Meixing-1	140	Zaotianhe
47	Cuijianali	94	Mantouyulvke	141	Zhanggongyuan

**Table 2:** Genetic diversity analysis of nutritional components of kernels in 141 apricot germplasm resources.

Nutritional Components	Mean	SD	CV %	Max	Min	Range	*H*′
Protein (PR) (g/100 g)	21.44	1.82	8.49	25.97	17.50	8.47	2.06
Amino acid ^1^ (AA) (g/100 g)	26.02	3.00	11.52	35.15	18.66	16.50	1.82
Fat (g/100 g)	40.71	7.82	19.21	51.85	16.64	35.21	2.01
Palmitic acid (PA) (g/100 g)	1.90	0.30	15.88	2.61	0.99	1.62	1.92
Oleic acid (OA) (g/100 g)	23.84	6.15	25.78	35.20	7.91	27.29	2.01
Linoleic acid (LA) (g/100 g)	12.76	1.85	14.51	17.10	8.40	8.70	2.08
Fatty acid ^2^ (FA) (g/100 g)	39.30	6.71	17.07	49.70	19.00	30.70	1.91
Vitamin B_1_ (VB) (mg/100 g)	0.41	0.11	26.59	0.83	0.17	0.66	1.78
γ-tocopherol (γt) (mg/100 g)	24.23	4.23	17.48	37.70	11.09	26.61	1.81
α-tocopherol (αT) (mg/100 g)	1.33	0.47	35.43	3.18	0.49	2.69	1.75
δ-tocopherol (δT) (mg/100 g)	1.25	0.43	34.73	2.88	0.44	2.44	1.81
Vitamin E (VE) (mg/100 g)	3.77	0.68	18.09	5.60	2.60	3.00	2.09
Zinc (Zn) (mg/kg)	63.89	11.68	18.28	111.05	39.11	71.95	1.76
Potassium (K) (g/kg)	9.69	3.06	31.55	22.25	4.26	17.99	1.77
Calcium (Ca) (g/kg)	1.43	0.34	24.18	3.14	0.79	2.35	1.52

^1^ Amino acid refers to the sum of 18 types of amino acids; ^2^ fatty acid refers to the sum of 12 types of fatty acids.

**Table 3:** Principal component analysis of 15 nutritional components in kernels of 141 apricot cultivars.

Nutritional Components	Principal Component				
	1	2	3	4	5
Fatty acids (g/100 g)	0.930	0.198	0.189	0.140	0.125
Oleic acid (g/100 g)	0.924	−0.0004	0.179	0.006	0.198
Fat (g/100 g)	0.923	0.181	0.15	0.172	0.126
Palmitic acid (g/100 g)	0.699	0.465	0.206	−0.009	−0.009
Vitamin E (mg/100 g)	−0.694	0.476	0.411	0.106	0.275
γ-tocopherol (mg/100 g)	−0.675	0.49	0.048	0.406	−0.061
Linoleic acid (g/100 g)	0.127	0.634	0.043	0.492	−0.212
δ-tocopherol (mg/100 g)	−0.429	0.565	−0.120	0.109	−0.305
Protein (g/100 g)	−0.288	−0.540	0.460	0.170	0.025
α-tocopherol (mg/100 g)	−0.427	0.265	0.593	−0.225	0.487
Amino acids (g/100 g)	−0.109	−0.389	0.580	0.262	−0.074
Potassium (g/kg)	−0.519	0.184	0.239	−0.544	−0.175
Zinc (mg/kg)	−0.319	−0.472	0.003	0.536	0.043
Vitamin B_1_ (mg/100 g)	0.177	−0.276	0.481	0.066	−0.532
Calcium (g/kg)	−0.317	−0.174	−0.396	0.308	0.438
Characteristic value	4.975	2.352	1.666	1.310	1.039
Contribution rate (%)	33.167	15.68	11.108	8.732	6.926
Accumulating contribution rate (%)	33.167	48.848	59.955	68.688	75.614

**Table 4:** Characteristics of nutritional components in apricot kernels from 141 apricot cultivars.

Nutritional Components	Index	Germplasm Groups				
		I	II	III	IV	V
Protein (g/100 g)	Mean	21.77 ± 1.63 ab	21.01 ± 1.89 b	21.71 ± 1.86 ab	24.48 ± 2.11 a	22.14 ± 1.31 ab
	CV%	7.51	8.99	8.57	8.64	5.9
Amino acids (g/100 g)	Mean	26.38 ± 3.05 ab	25.73 ± 3.00 b	26.46 ± 2.35 ab	30 ± 3.70 a	24.65 ± 3.27 ab
	CV%	11.56	11.65	8.9	12.34	13.26
Fat (g/100 g)	Mean	42.21 ± 4.18 b	45.13 ± 3.59 a	32.52 ± 3.84 c	23.48 ± 3.21 c	25.42 ± 4.41 c
	CV%	9.9	7.95	11.79	13.66	17.36
Palmitic acid (g/100 g)	Mean	1.85 ± 0.17 b	2.06 ± 0.25 a	1.69 ± 0.20 bc	1.21 ± 0.00 c	1.33 ± 0.24 c
	CV%	9.14	12.13	11.57	0	18.08
Oleic acid (g/100 g)	Mean	24.15 ± 4.14 b	27.34 ± 3.4 a	15.67 ± 3.12 c	11.49 ± 2.70 c	10.53 ± 1.80 c
	CV%	17.16	12.44	19.87	23.51	17.12
Linoleic acid (g/100 g)	Mean	13.13 ± 1.66 a	12.72 ± 1.90 a	12.73 ± 1.40 a	9.21 ± 0.04 b	11.26 ± 1.90 ab
	CV%	12.64	14.96	11.02	0.46	16.89
Fatty acids (g/100 g)	Mean	39.93 ± 3.89 b	42.97 ± 3.13 a	30.79 ± 4.20 c	22.4 ± 2.83 c	23.7 ± 3.87 c
	CV%	9.73	7.29	13.64	12.63	16.34
Vitamin B_1_ (mg/100 g)	Mean	0.44 ± 0.12 a	0.41 ± 0.10 a	0.41 ± 0.15 a	0.33 ± 0.12 a	0.33 ± 0.07 a
	CV%	26.92	23.46	35.84	35.94	21.02
γ-tocopherol (mg/100 g)	Mean	24.68 ± 3.59 b	22.46 ± 3.02 c	28.97 ± 4.49 a	24.84 ± 1.75 abc	27.72 ± 5.52 ab
	CV%	14.55	13.46	15.49	7.04	19.9
α-tocopherol (mg/100 g)	Mean	1.27 ± 0.47 a	1.24 ± 0.34 a	1.52 ± 0.42 a	2.15 ± 1.45 a	1.53 ± 0.48 a
	CV%	36.87	27.16	27.7	67.19	31.55
δ-tocopherol (mg/100 g)	Mean	1.26 ± 0.44 ab	1.13 ± 0.34 b	1.45 ± 0.40 a	1.71 ± 0.27 a	1.4 ± 0.68 ab
	CV%	34.67	30.03	27.67	16.06	48.46
Vitamin E (mg/100 g)	Mean	3.75 ± 0.67 bc	3.5 ± 0.50 c	4.43 ± 0.62 a	4.66 ± 1.27 ab	4.31 ± 0.75 ab
	CV%	17.74	14.17	14.07	27.28	17.37
Zinc (mg/kg)	Mean	72.94 ± 7.02 a	56.02 ± 6.78 b	60.74 ± 7.39 b	51.63 ± 5.30 b	82.53 ± 5.36 a
	CV%	9.63	12.1	12.17	10.27	6.5
Potassium (g/kg)	Mean	8.76 ± 2.35 b	9.09 ± 2.57 b	11.98 ± 2.39 b	21.16 ± 1.54 a	10.91 ± 2.24 b
	CV%	26.85	28.24	19.97	7.26	20.55
Calcium (g/kg)	Mean	1.42 ± 0.27 ab	1.34 ± 0.27 b	1.46 ± 0.31 ab	1.13 ± 0.04 b	1.88 ± 0.50 a
	CV%	19.35	19.86	20.89	3.41	26.44

Note: Different small letters in the same line indicate significant differences (*p* < 0.05).

**Table 5:** Factor scores of the top 20 apricot cultivars for kernel nutrition ranked by comprehensive evaluation.

Cultivar	F1	F2	F3	F4	F5	D	Ranking
Kuhehonglian	2.15	2.10	1.27	0.87	0.17	1.68	1
Machuanling	2.33	1.42	1.40	0.80	0.16	1.63	2
Guajiayutianhexianbai	2.43	0.57	1.47	1.02	0.12	1.53	3
Guanlaoyelian	2.11	0.97	1.46	1.24	0.30	1.51	4
GRAFLTHAN	2.50	0.25	1.42	0.78	0.30	1.47	5
Xingtaihongjiexing-1	2.12	1.22	1.12	0.77	0.12	1.45	6
Dapiantou	2.04	1.19	1.19	0.99	0.11	1.44	7
Chuanzhihong	2.10	0.62	1.54	1.10	0.30	1.43	8
Xifuxing	1.93	1.28	1.16	1.09	0.22	1.43	9
Caopixing	2.10	0.99	1.43	0.65	0.08	1.42	10
Dayushisheng	1.99	0.95	1.19	1.10	0.40	1.41	11
Yangshaohuang No.2	1.91	1.15	1.39	0.92	0.15	1.40	12
JITKA	1.75	1.02	1.74	1.16	0.11	1.38	13
Xingtaihongjiexing-2	2.10	1.00	1.06	0.68	0.12	1.37	14
Meixing-2	2.15	0.52	1.50	0.57	0.34	1.37	15
Badou	1.88	1.02	1.26	0.90	0.24	1.35	16
99-23-1	1.76	1.16	1.58	0.77	0.16	1.35	17
Bergeron	1.90	0.94	1.34	0.86	0.20	1.34	18
Yangshaohung No.1	1.67	0.99	1.63	1.18	0.17	1.33	19
Mantouyulvke	1.62	1.04	1.59	1.22	0.33	1.33	20

## Data Availability

The original contributions presented in this study are included in the article/Supplementary Materials. Further inquiries can be directed to the corresponding author.

## Supplementary Materials

The following supporting information can be downloaded at: https://www.mdpi.com/article/10.3390/foods15203588/s1, Table S1: The composition information of essential amino acids in apricot kernels from 141 cultivars; Table S2: The composition information of fatty acids in apricot kernels from 141 cultivars.
