# Supplementary material for: Comprehensive Nutritional Profiling of Kernels in 141 Apricot Cultivars Based on Amino Acids, Fatty Acids, Vitamins, and Mineral Elements
Source: Foods. 2026 Oct 9;15(20):3588. doi: 10.3390/foods15203588 (PMC13703220; doi:10.3390/foods15203588)
Supplement: Supplementary file 1 [file foods-15-03588-s001.zip › foods-4582886-supplementary.pdf]

Table S1 The composition information of essential amino acids in apricot kernels from 141 cultivars

Index	Amino acid composition								Sum
	THR	VAL	MET	ILE	LEU	PHE	LYS	Trp	
Minimum (g/100g)	0.39	**0.78**	0.17	0.69	**1.36**	**1.03**	0.57	0.11	**5.37**
Maximum (g/100g)	0.78	**1.53**	0.52	1.36	**2.81**	**1.95**	1.05	0.25	**9.67**
Mean (g/100g)	0.61	**1.08**	0.31	0.96	**1.91**	**1.49**	0.76	0.19	**7.31**
CV%	11.08	11.87	**28.68**	11.63	12.32	12.62	10.36	12.32	10.45

Table S2 The composition information of fatty acids in apricot kernels from 141 cultivars

Index	Fatty acid composition														
	C14:0	C16:0	C17:0	C18:0	C20:0	C22:0	C24:0	**Saturated fatty acid (SAF)**	C16:1n7	C18:1n 9c	C18:2n 6c	C18:3n 3	C20:1	**Unsaturated fatty acid (USAF)**	**USAF/ASF (%)**
	Myristic acid	Palmitic acid	Margaric acid	Stearic acid	Arachidic acid	Behenic acid	Lignoceric acid		Palmitoleic acid	Oleic acid	Linoleic acid	α-Linolenic acid	cis-11-Eicosenoic acid		
Min (g/100g)	0.005	0.989	0.007	0.148	0.019	0.007	0.008	**1.237**	0.121	7.910	8.400	0.019	0.0123	**17.413**	**10.46**
Max (g/100g)	0.054	2.610	0.021	0.691	0.054	0.012	0.016	**3.328**	0.424	35.200	16.700	0.0687	0.0391	**46.835**	**21.03**
Mean (g/100g)	0.016	1.894	0.014	0.430	0.033	0.009	0.011	**2.378**	0.258	23.741	12.725	0.0330	0.0254	**36.781**	**15.50**
CV%	69.20	15.89	21.60	24.92	19.41	13.08	16.02	**16.28**	23.02	26.17	14.51	19.75	23.81	**17.59**	**12.22**
